# Adverse Events Related to Tirzepatide

**DOI:** 10.1210/jendso/bvad016

**Published:** 2023-01-26

**Authors:** Rahul Mishra, Rishi Raj, Ghada Elshimy, Isain Zapata, Lakshmi Kannan, Priyanka Majety, Dinesh Edem, Ricardo Correa

**Affiliations:** Department Hematology and Oncology, Cleveland Clinic Foundation, Cleveland, Ohio 44195, USA; Department of Endocrinology, Diabetes, and Metabolism, Pikeville Medical Center, Pikeville, Kentucky 41501, USA; Department of Endocrinology, Diabetes, and Metabolism, Medical College of Georgia, Augusta University, Augusta, Georgia 30912, USA; Department of Biomedical Sciences, Rocky Vista University, Parker, Colorado 80112, USA; Department of Nephrology, Pikeville Medical Center, Pikeville, Kentucky 41501, USA; Division of Endocrinology, Diabetes and Metabolism, Virginia Commonwealth University, Richmond, Virginia 23298, USA; Department of Endocrinology and Diabetes, University of Arkansas for Medical Sciences, Little Rock, Arkansas 72205, USA; Department of Endocrinology, Diabetes and Metabolism, Institute of Endocrinology and Metabolism, Cleveland Clinic, Cleveland, Ohio 44195, USA

**Keywords:** LY3298176, dual GIP/GLP-1 receptor agonist, tirzepatide, safety, adverse events, diabetes, glucose, systematic review, meta-analysis

## Abstract

**Context:**

Tirzepatide is a dual glucose-dependent insulinotropic peptide (GIP) and glucagon-like peptide-1 receptor agonist (GLP-1 RA) approved by the US Food and Drug Administration in May 2022 for patients with type 2 diabetes mellitus (T2DM).

**Objective:**

We aimed to determine the rates of individual adverse events (AEs) related to 3 studied doses of tirzepatide.

**Methods:**

We performed a systematic review with meta-analysis including 5 databases (PubMed, Embase, CINAHL, Scopus, and Web of Science) for all clinical trials reporting AEs related to tirzepatide. The safety data from individual studies were extracted and analyzed through meta-regression to assess rates of individual AEs. Study quality assessment was performed using the National Heart, Lung, and Blood Institute Quality Assessment Tool for Observational Cohort and Cross-Sectional Studies.

**Results:**

Ten trials (6836 participants) were included. Gastrointestinal (GI) AEs were the most commonly reported AEs and were dose dependent 39% (95% CI, 35%-43%), 46% (95% CI, 42%-49%), and 49% (95% CI, 38%-60%) for the 5, 10, and 15 mg dose, respectively. Among all GI AEs, nausea and diarrhea were most frequent at any dose of tirzepatide. Drug discontinuation due to AEs was highest with the 15 mg dose of tirzepatide (10%). Incidence of mild hypoglycemia (blood glucose < 70 mg/dL) was highest with tirzepatide 10 mg dose 22.6% (9.2%-39.8%). Rates of fatal AEs, severe hypoglycemia, acute pancreatitis, cholelithiasis, and cholecystitis were extremely low (≤ 1%) across all doses of tirzepatide.

**Conclusion:**

Tirzepatide is associated with a dose-dependent increase in incidence of GI AEs and AEs leading to drug discontinuation. Severe hypoglycemia, fatal AEs, acute pancreatitis, cholelithiasis, and cholecystitis are rare with this medication.

The term “*incretin*” was first described in 1932, referring to the hormones released from the gut that regulate the insulin response to a meal [1]. It is also known as a glucose-dependent insulinotropic polypeptide (GIP), which lowers the glucagon level by increasing the release of insulin in response to glucose ingestion. Elrick et al [2] first demonstrated a significant and sustained rise in plasma insulin response following an oral glucose load compared to a parental glucose load. This phenomenon was later known as “the incretin effect” and accounts for up to 65% of postprandial insulin secretion [3]. The 2 principal incretin hormones responsible for the incretin effect are glucagon-like peptide 1 (GLP-1) and GIP.

GLP-1 acts by stimulating insulin release and inhibiting glucagon secretion in the hyperglycemic and normoglycemic states. Extra pancreatic effects of GLP-1 include delayed gastric emptying, increased satiety, and reduced food intake leading to reduction in body weight and glycated hemoglobin A_1c_ (HbA_1c_) in patients with type 2 diabetes mellitus (T2DM) [4-6]. GLP-1 receptor agonists (GLP-1 RAs) have been approved for the treatment of T2DM since 2005 and are recommended, as per the latest guidelines, early in the treatment algorithm given the associated weight reduction, glycemic efficacy, and favorable cardio-nephrovascular outcomes [7, 8].

GIP also stimulates glucose-dependent insulin secretion and is responsible for a greater proportion of the incretin effect than GLP-1. It differs from GLP-1 in its effect on glucagon secretion: It has a glucagonotropic property in the normoglycemic and hypoglycemic state, and glucagonostatic in the hyperglycemic state [9-11]. It also enhances the sensitivity of adipose tissue to insulin and the postprandial lipid-buffering capacity of white adipose tissue [12, 13]. Although it was thought to have no potential as a glucose-lowering therapy because of observations showing no insulinotropic effect from supraphysiological infusion in people with T2DM, recent evidence showed that there is a synergistic effect of GLP-1 and GIP, resulting in significantly increased insulin response and glucagonostatic response [10]. This led to the development of the dual GIP/GLP-1 RA.

Tirzepatide was recently approved by the US Food and Drug Administration in May 2022 for the treatment of T2DM. In addition to the 39 amino acids, it has a 20-carbon fatty diacid moiety that has the capacity to bind to albumin, prolonging its half-life to 5 days, allowing once-weekly dosing. It has a comparable GIP receptor binding affinity to native GIP. However, it has a 5 times lower GLP-1 receptor affinity than that of native GLP-1 [14, 15]. The SURPASS clinical trial programs assessed the efficacy and safety of tirzepatide at 3 different doses (5, 10, and 15 mg) in patients with T2DM. The recommended dose-escalation algorithm is to start at a dose of 2.5 mg weekly for the first 4 weeks followed by increments of 2.5 mg every 4 weeks until the maintenance dose based on the maximum tolerated side effects [15]. The SURPASS-5 trials showed that diarrhea, nausea, and vomiting were the most common treatment-emergent adverse events (AEs) in the groups receiving tirzepatide; however, the incidence of new AEs decreased over time in the groups and there were no deaths were recorded during the study [16]. Since this drug was recently approved for use, it is of considerable importance to assess the rate of AEs reported in different clinical trials. This meta-analysis is aimed to assess pooled rates of AEs associated with the use of tirzepatide at 5, 10 or 15 mg doses.

## Materials and Methods

### Study Design and Selection Criteria

This systematic review and meta-analysis was performed using the guidelines established by the Preferred Reporting Items for Systematic Reviews and Meta-Analyses statements (PRISMA) [17]. We included all the clinical trials reporting any AE related to tirzepatide published before March 12, 2022. We excluded the following studies: (a) preclinical studies, and (b) nonclinical trial publications such as comments, reviews, editorials, opinions, etc. The outcomes of interest in this study consisted of 3 overall safety outcomes and other specific outcomes. The overall safety outcomes were any serious AEs, any AEs leading to drug discontinuation, and any AEs leading to death. The specified safety outcomes were gastrointestinal (GI) AEs, gall bladder and pancreatic AEs, appetite and weight changes, hypoglycemia, severe hypoglycemia, allergic reactions, and multisystem AEs. Given that this drug was recently approved and has a limited number of randomized controlled trials available, we also included data from a phase 1 clinical trial in our meta-analysis [18].

### Search Strategy

**Figure 1.**
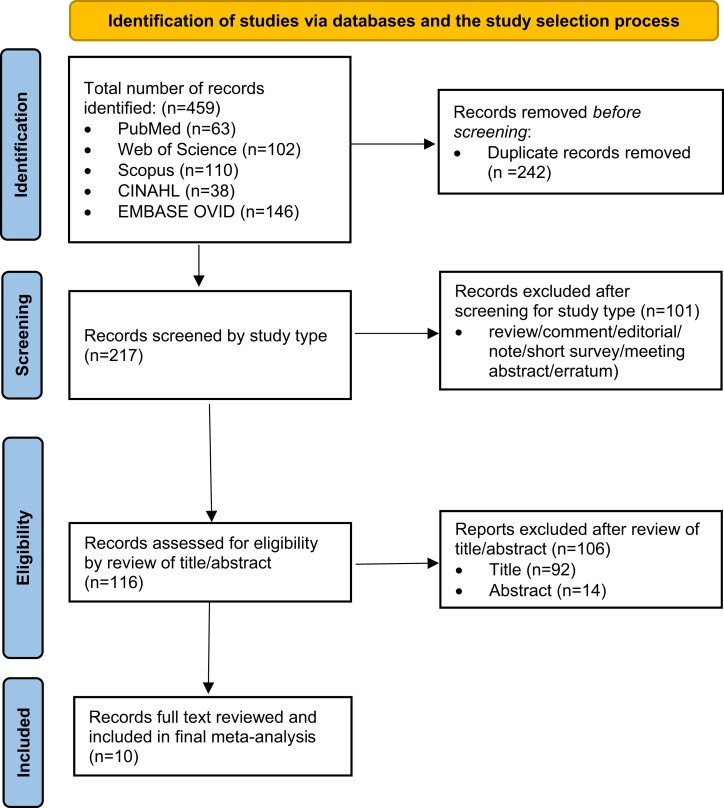
PRISMA (Preferred Reporting Items for Systematic Reviews and Meta-Analyses) flow diagram of the study selection process.

We conducted a comprehensive search of the following 5 databases: (a) PubMed/Medline, (b) Embase, (c) CINAHL, (d) Scopus, and (e) Web of science. We restricted our search to articles published in the English language. The search strategy was designed by following the Population, Intervention, Comparison, and Outcome (PICO) Principle. Using our methodology, the following key words were identified: “Tirzepatide” OR “LY3298176” OR “LY-3298176.” Next, the bibliographies of the selected articles were manually searched for any additional studies. After screening for duplicate studies, 2 reviewers (R.R. and R.M.) independently reviewed the title and abstract of the identified publications. We then excluded studies that did not address our research question based on our prespecified inclusion/exclusion criteria. Finally, the full texts of the remaining articles were examined to determine the final exclusion for our systematic review. Any conflicts during the study selection process were resolved by the 2 reviewers by thorough discussion. Fig. 1 shows the schematic diagram of the study selection process.

### Data Extraction

Data were extracted from each eligible study included in the meta-analysis: study title, primary author, year of publication, sample size, patient demographics study details (single vs multicenter), study duration, average HbA1c, duration of diabetes, and tirzepatide dose assessed. Table 1 summarizes the details of the studies included in the meta-analysis.

**Table 1.** Baseline characteristics of included studies

Author	Tirzepatide doses, mg	Control arm	Control arm (N)	Tirzepatide arm (N)	Mean age, y	Female N (%)	Mean diabetes, duration y	Mean baseline A_1c_, gm%
Frías, 2018	1, 5, 10, 15	Placebo, dulaglutide	105	211	57	148 (47)	9	8.1
Coskun, 2018	Incremental doses	Placebo	11	42	56.8	25 (47)	NA	8.4
Frías, 2020	Incremental doses	Placebo	26	85	57.4	45 (40)	9.1	8.4
Rosenstock, 2021	5, 10, 15	Placebo	115	363	54.1	231 (48)	4.7	7.9
Urva, 2021	5	NA	NA	45	60.3	15 (33)	NA	NA
Ludvik, 2021	5, 10, 15	Insulin (degludec)	360	1077	57.4	635 (44)	8.4	8.17
Del Prato, 2021	5, 10, 15	Insulin (glargine)	1000	995	63.6	749 (38)	11.8	8.52
Frías, 2021	5, 10, 15	Placebo	469	1409	56.6	996 (53)	8.6	8.28
Furihata, 2022	Incremental doses	Placebo	9	39	57.4	1 (2)	8.5	8
Dahl, 2022	5, 10, 15	Placebo	120	355	60.6	211 (44)	13.3	8.3

Abbreviation: NA, not available.

### Data Synthesis

Compiled data were summarized for completion and evaluated through meta-regression by analysis of variance (ANOVA). To perform this, we performed first a meta-analysis on event proportions done per parameter evaluated (32 parameters) across all tirzepatide doses (3 doses and the placebo). Owing to low availability of replication studies, the 12, 1, and 0.5 mg doses were dropped from the analysis; therefore, we considered only the more popular 15, 10 and 5 mg doses of tirzepatide and the placebo when reported. All initial meta-analysis were performed using MedCalc v.20.106 (MedCalc Software Ltd) using the Meta-analysis: proportion function. Because of variations in heterogeneity across all parameters and doses evaluated, only random-effect models were further used in the analysis.

Second, we performed meta-regression and ANOVA using SAS/STAT v9.4 (SAS Institute Inc) with the use of generalized linear models (GLMs) through PROC GLM, where the weights estimated for each study in the initial meta-analysis were used as the weights for the meta-regression. Differences between doses groups (15, 10, and 5 mg tirzepatide) were evaluated along with the placebo group. Associations were declared statistically significant when the probability of the ANOVA test was significant after a multiple testing adjustment (0.05/32 tests, using the Bonferroni approach).

### Quality Assessment

Two independent reviewers (R.R. and R.M.) assessed the study quality and risk of bias in the included studies using the National Heart, Lung, and Blood Institute Quality Assessment Tool for Observational Cohort and Cross-Sectional Studies (Study Quality Assessment Tools; https://www.nhlbi.nih.gov/health-topics/study-quality-assessment-tools). Any disagreements were resolved by discussing with other reviewers (G.E. and L.K.). Based on 14 questions, an overall rating was assigned as good, fair, and poor for study quality, corresponding to low, moderate, or high risk of bias.

## Results

### Eligible Studies and Characteristics

A total of 459 studies were retrieved through the search of databases (PubMed 63; Web of Science 102; Scopus 110; CINAHL 38; Embase 146). After removing duplicates, we obtained 217 records. Of these, 101 studies were excluded based on the study types and the remaining 116 studies were selected for screening by title and abstract. Ten studies were included for full-text assessment, all of which met the inclusion criteria for the study. The study flowchart is shown in Fig. 1. The 10 studies involving a total of 6836 adult participants were published between 2018 to 2022, and the baseline characteristics of the included studies are shown in Table 1. Mean age, diabetes duration, HbA_1c_, and number of females were 58.9 years, 9.6 years, 8.3 gm%, and 44.7% (n = 3056). Control and tirzepatide groups had 2215 and 4621 participants, respectively. Data from one study by Frias et al [19], in which participants received incremental doses of tirzepatide with a peak dose of 15 mg (2.5, 7.5, and 15 mg during 0-3, 4-7, and 8-11 weeks), was combined with a 15-mg group for analysis of AEs. In another study by Coskun et al [14], there was titration of tirzepatide doses until peak of 10 and 15 mg for respective group participants. Table 2 summarizes AEs seen with tirzepatide use. Using the study quality assessment tool, 9 studies were rated “good,” and 1 study was rated “fair”; none was rated “poor,” eliminating increased risk of bias in the studies included in our meta-analysis (Supplementary File 1) [20].

**Table 2.** Adverse events summary

	Placebo		Tirzepatide, mg						
			5		10		15		
AEs	Studies (sample size)	Proportion (95% CI)	Studies (sample size)	Proportion (95% CI)	Studies (sample size)	Proportion (95% CI)	Studies (sample size)	Proportion (95% CI)	ANOVA *P*
**Severity graded AEs**									
Serious AEs	NA	NA	6 (1458)	7.52 (4.85-10.72)	7 (1460)	7.32 (3.90-11.72)	8 (1501)	5.57 (3.15-8.62)	.773
Fatal AEs	NA	NA	5 (1333)	1.07 (0.14-2.88)	5 (1329)	0.72 (0.34-1.25)	6 (1369)	0.90 (0.33-1.75)	.688
AEs leading to treatment discontinuation	NA	NA	6 (1449)	7.23 (5.20-9.56)	6 (1448)	8.68 (7.29-10.18)	7 (1489)	10.39 (7.978-13.09)	.248
**GI AEs**									
All GI AEs^a^	2 (166)	15.46 (7.81-25.11)	3 (646)	39.05 (35.33-42.83)	3 (641)	45.57 (41.74-49.43)	3 (644)	49.25 (38.44-60.08)	.**004**
Nausea^a^	6 (332)	4.94 (2.89-7.50)	9 (1514)	13.27 (10.79-15.97)	8 (1472)	17.89 (14.91-21.08)	9 (1517)	24.08 (20.21-28.13)	**< **.**001**
Diarrhea^a^	6 (332)	8.32 (5.62-11.49)	9 (1514)	13.19 (10.89-15.69)	8 (1472)	17.22 (15.28-19.25)	9 (1517)	20.79 (16.20-25.80)	**< **.**001**
Vomiting^a^	6 (332)	2.73 (1.26-4.73)	9 (1514)	5.67 (4.57-6.89)	8 (1472)	8.32 (6.38-10.50)	9 (1517)	13.98 (9.62-19.01)	**< **.**001**
Dyspepsia^a^	6 (332)	2.16 (0.90-3.99)	8 (1469)	5.97 (4.65-7.46)	8 (1472)	8.52 (6.62-10.65)	9 (1517)	6.79 (5.31-8.44)	**< **.**001**
Abdominal distension	3 (71)	3.76 (0.66-9.25)	4 (120)	8.28 (2.01-18.25)	3 (75)	11.95 (5.74-20.02)	3 (81)	16.29 (0.27-49.36)	.471
Constipation	5 (321)	1.54 (0.49-3.16)	6 (1102)	6.07 (4.75-7.56)	6 (1100)	8.86 (4.67-14.22)	7 (1146)	7.57 (4.30-11.67)	.476
Abdominal discomfort	2 (60)	4.36 (0.74-10.80)	2 (66)	2.52 (0.16-7.54)	2 (63)	7.89 (0.01-28.07)	2 (69)	14.86 (2.43-35.18)	.139
Abdominal pain	2 (37)	4.24 (0.26-12.70)	3 (837)	2.62 (1.65-3.81)	3 (841)	4.64 (3.33-6.16)	4 (869)	7.61 (4.34-11.72)	.251
Eructation	NA	NA	2 (125)	5.27 (2.08-9.82)	2 (131)	4.35 (1.46-8.68)	2 (132)	16.42 (0.25-49.86)	.756
**Gall bladder and pancreatic AEs**									
Cholelithiasis^a^	2 (235)	0.21 (0.03-1.198)	5 (1394)	0.95 (0.51-1.52)	5 (1397)	0.53 (0.22-0.97)	5 (1408)	0.52 (0.21-0.97)	.**008**
Cholecystitis	2 (77)	0.62 (0.10-3.54)	3 (742)	0.09 (0.00-0.43)	3 (739)	0.55 (0.01-1.89)	4 (778)	0.24 (0.02-0.71)	.359
Acute pancreatitis	5 (323)	0.33 (0.01-1.24)	7 (1458)	0.39 (0.05-1.07)	7 (1460)	0.36 (0.12-0.74)	8 (1501)	0.32 (0.10-0.67)	.527
Increased lipase^b^	3 (180)	2.28 (0.62-4.93)	4 (511)	3.58 (2.15-5.36)	4 (510)	3.88 (1.75-6.82)	4 (527)	6.86 (4.87-9.17)	.**019**
**Appetite and weight**									
Decreased appetite	6 (332)	1.97 (0.77-3.73)	8 (1469)	11.06 (7.01-15.90)	8 (1472)	13.64 (9.37-18.57)	9 (1517)	19.38 (13.01-26.67)	.106
Decreased weight	NA	NA	2 (64)	4.11 (0.67-10.18)	2 (63)	12.09 (5.36-21.06)	2 (65)	8.91 (0.63-25.25)	.195
**Hypoglycemic AEs**									
Hypoglycemia (plasma glucose ≤ 70 mg/dL	NA	NA	6 (988)	17.39 (4.73-35.77)	6 (991)	22.6 (9.18-39.83)	7 (1031)	20.99 (8.85-36.68)	.937
Hypoglycemia (plasma glucose ≤ 54 mg/dL)	3 (261)	3.91 (0.01-14.53)	5 (1394)	3.64 (0.56-9.27)	5 (1397)	3.29 (0.34-9.12)	6 (1436)	3.49 (0.95-7.56)	.998
Severe hypoglycemia	5 (323)	0.33 (0.01-1.24)	7 (1458)	0.25 (0.06-0.58)	7 (1460)	0.17 (0.02-0.50)	8 (1501)	0.54 (0.23-1.05)	.197
**Respiratory AEs**									
Upper respiratory tract infection	NA	NA	2 (66)	7.14 (2.28-14.41)	2 (63)	5.85 (1.50-12.80)	2 (69)	3.82 (0.65-9.49)	.496
Nasopharyngitis	NA	NA	5 (979)	6.05 (2.85-10.34)	5 (979)	5.07 (3.79-6.53)	5 (991)	6.42 (4.07-9.27)	.762
Influenza	NA	NA	2 (176)	5.57 (2.69-9.41)	2 (172)	4.9 (0.98-11.58)	2 (174)	0.89 (0.04-4.34)	.287
**Allergic reaction**									
Injection site reaction	5 (323)	1.15 (0.24-2.73)	7 (1458)	1.89 (0.75-3.54)	7 (1460)	2.44 (1.34-3.85)	8 (1501)	3.11 (1.47-5.34)	.229
Hypersensitivity	4 (312)	3.88 (1.14-8.17)	5 (1120)	3.23 (1.92-4.86)	5 (1120)	3.03 (2.11-4.12)	6 (1151)	2.42 (1.48-3.58)	.751
**Multisystem AEs**									
Headache	NA	NA	5 (241)	4.00 (1.92-6.80)	4 (196)	5.16 (1.10-11.96)	5 (230)	10.71 (4.84-18.54)	.429
Hypertension	NA	NA	3 (529)	2.61 (1.22-4.51)	3 (530)	2.04 (1.01-3.41)	3 (532)	2.61 (1.43-4.14)	.755
Diabetic retinopathy	NA	NA	3 (1157)	0.59 (0.02-1.91)	3 (1157)	0.57 (0.04-1.71)	3 (1167)	0.43 (0.02-1.40)	.935
MACE	NA	NA	2 (474)	0.73 (0.17-1.70)	2 (479)	1.02 (0.32-2.12)	2 (479)	0.56 (0.09-1.43)	.935
Malignant neoplasms	NA	NA	2 (474)	1.21 (0.43-2.39)	2 (479)	1.44 (0.57-2.70)	2 (479)	0.72 (0.16-1.69)	.485

Abbreviations: AE, adverse event; ANOVA, analysis of variance; GI, gastrointestinal; MACE, major adverse cardiovascular events; NA, not available.

a On comparing with placebo, the proportion of AEs for all 3 doses (5, 10, and 15 mg) of tirzepatide had a *P* value less than or equal to .05 (Depicted in bold in Table 2).

b On comparing with placebo, the proportion of AEs had a *P* value less than or equal to .05 only for the 15 mg dose of tirzepatide.

### Severity Graded Adverse Events

**Figure 2.**
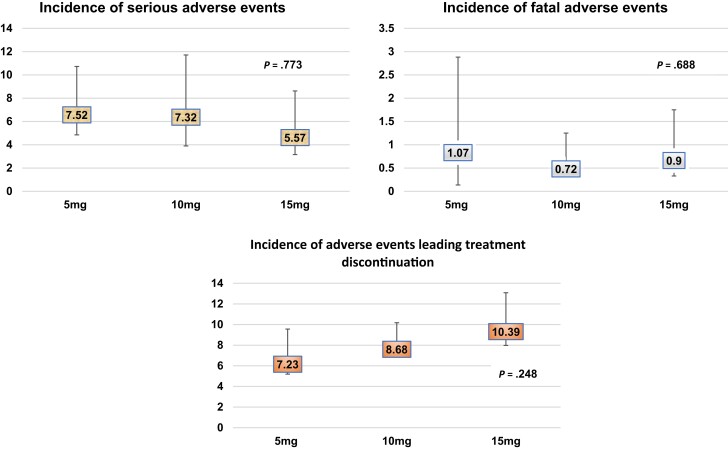
Incidence (%) of serious adverse events, fatal adverse events, and adverse events leading to treatment discontinuation with tirzepatide 5, 10, and 15 mg, respectively.

Specific AEs were reported by a variable number of studies. The proportion of serious AEs was 7.52% with 5 mg and about 7.32% with 10 mg and 5.57% with 15-mg doses of tirzepatide. The 15-mg dose had the highest proportion of patients with AEs leading to treatment discontinuation. The proportion of fatal AEs was 1.07% with 5 mg, below 0.72% with 10, and 0.90% with 15-mg doses. ANOVA *P* values were not statistically significant for any of these AEs among different doses. Severity grade AEs are also represented in Fig. 2.

### Incidence of Gastrointestinal Adverse Events

GI AEs were reported by most of the studies included in the meta-analysis. “All GI AEs” were reported in 2 studies [14, 21] for placebo and 3 studies [21-23] for the tirzepatide arm. Three studies reported all GI AEs with tirzepatide. A significantly higher proportion of patients experienced GI AEs with tirzepatide, and the pooled proportion for all GI AEs were 39.05% (95% CI, 35.33%-42.83%), 45.57% (95% CI, 41.74%-49.43%), and 49.25% (95% CI, 38.44%-60.08%) for the 5, 10, and 15 mg dose, respectively. Among all GI AEs, nausea and diarrhea were the most frequently reported AEs with any doses of tirzepatide. Individual GI AEs such as nausea, vomiting, diarrhea, and dyspepsia were reported by a variable number of studies. Nine studies [14, 16, 18, 21-26] reported rates of nausea, vomiting, diarrhea, and dyspepsia. One study [18] did not report rates of nausea, vomiting, and diarrhea with the 10-mg dose of tirzepatide. Data for dyspepsia from Frias 2020 [19] were analyzed with the 15-mg dose, as mentioned earlier. The pooled proportion of nausea was 13.27% (95% CI, 10.79%-15.97%), 17.89% (95% CI, 14.91%-21.08%), and 24.08% (95% CI, 20.21%-28.13%) for the 5-, 10-, and 15-mg dose, respectively. Similar rates of diarrhea were seen with tirzepatide and were 13.19%, 17.22%, and 20.79% for the 5-, 10-, and 15-mg dose, respectively. Vomiting was less frequent with pooled proportions of 5.67%, 8.32%, and 13.98% with the 5-, 10-, and 15-mg dose, respectively. The proportion of any GI AEs increased with increasing doses of tirzepatide. Surprisingly, dyspepsia and constipation were most frequently seen with the 10-mg dose of tirzepatide, and the rates were 8.52% (95% CI, 6.62%-10.65%) and 8.86% (95% CI, 4.67%-14.22%), respectively. The pooled proportion of abdominal distension, abdominal discomfort, eructation, and abdominal pain was highest with the 15-mg dose of tirzepatide (16.29%, 14.86%, 16.42% and 7.57%, respectively).

### Incidence of Gallbladder and Pancreatic Adverse Events

Cholelithiasis was reported in 5 studies [16, 21, 23-25]. The pooled proportion was nonsignificant at 0.95% (95% CI, 0.51%-1.52%) with the 5-mg dose and decreased further with the 10- and 15-mg doses. The pooled proportion of cholecystitis was 0.01% (95% CI, 0.00%-0.43%), 0.55% (95% CI, 0.01%-1.89%), and 0.24% (95% CI, 0.02%-0.71%) for the 5-, 10-, and 15-mg dose, respectively. Seven studies [14, 16, 21-25] reported acute pancreatitis with the 5- and 10-mg dose while 8 studies reported acute pancreatitis with the 15 mg dose. The pooled proportion for acute pancreatitis was 0.39% (95% CI, 0.05%-1.07%), 0.36% (95% CI, 0.12%-0.74%), and 0.32% (95% CI, 0.10%-0.67%) for the 5-, 10-, and 15-mg dose, respectively. Five studies [14, 16, 19, 21, 22] with a placebo arm had a similar pooled proportion for acute pancreatitis of 0.33% (95% CI, 0.01%-1.24%). Four studies reported increased lipase levels with a pooled proportion of 3.58% (95% CI, 2.15%-5.36%), 3.88% (95% CI, 1.75%-6.82%), and 6.86% (95% CI, 4.87%-9.17%) for the 5-, 10-, and 15-mg dose, respectively. The pooled proportion of lipase was highest with the 15 mg dose. Additionally, the pooled proportion of elevated lipase was higher with any dose of tirzepatide compared to placebo (pooled proportion of 2.28% [95% CI, 0.62%-4.93%], reported in 3 studies [16, 22, 26] with a placebo arm).

### Incidence of Hypoglycemia

The incidence of plasma glucose less than or equal to 70 mg/dL and less than or equal to 54 mg/dL were reported by 6 [14, 16, 21, 22, 24, 25] and 5 studies [16, 21, 23-25] respectively for different doses of tirzepatide. Data from one study [19] were considered only for the 15-mg dose for all hypoglycemic AEs. The proportion of hypoglycemic events did not differ significantly with different dose of tirzepatide. The pooled proportion (95% CI) of glucose levels less than or equal to 70 mg/dL was at maximum with 10 mg (22.6% [9.18%-39.83%]), followed by the 15-mg (20.99% [8.85%-36.68%]) and 5-mg dose (17.39% [4.73%-35.77%]), respectively. The pooled proportion (95% CI) of glucose levels less than 54 mg/dL was nearly the same at all 3 doses (3.64% [0.56%-9.27%] for 5 mg, 3.29% [0.34%-9.12%] for 10 mg, and 3.49% [0.95%-7.56%] for the 15-mg dose of tirzepatide). Three studies reported an incidence of 3.91% (0.01%-14.53%) for blood glucose less than or equal to 54 mg/dL in the placebo group. The pooled incidence of severe hypoglycemia (defined by an event during which the patient requires the assistance of another person to actively administer carbohydrate, glucagon, or other resuscitative actions) was reported among 5 studies [14, 16, 19, 21, 22] for placebo, and 7 studies [14, 16, 21-25] for different doses of tirzepatide. The incidence rate (95% CI) of severe hypoglycemia was 0.33% (0.01-1.24) for placebo 0.25% (0.06-0.58) for 5 mg, 0.17% (0.02-0.50) for 10 mg, and 0.54% (0.23-1.05) for the 15-mg dose of tirzepatide.

### Incidence of Other Adverse Events

Injection site reaction was reported by 5 studies for placebo [14, 16, 19, 21, 22], 7 studies [14, 16, 21-25] at 5 and 10 mg, and 8 studies (including [19]) for the 15-mg dose of tirzepatide. Pooled proportion (95% CI) of injection site reaction was 1.15% (0.24%-2.73%) with placebo, 1.89% (0.75%-3.54%) with 5 mg, 2.44% (1.34%-3.85%) with 10 mg, and 3.11% (1.47%-5.34%) with the 15-mg dose of tirzepatide. Hypersensitivity reaction was assessed by 4 studies [16, 19, 21, 22] for a placebo group (3.88% [1.14%-8.17%]), 5 studies [16, 21-23, 25] for 5 mg (3.23% [1.92%-4.86%]), and 10 mg (3.03% [2.11%-4.12%]) doses, and 6 studies [16, 19, 21-23, 25] for 15 mg (2.42% (1.48%-3.58%]). Among respiratory AEs, nasopharyngitis was assessed in 5 studies [16, 21, 22, 24, 25], while upper respiratory tract infection [23, 26] and influenza [21, 23] were assessed in 2 studies each. Incidence (95% CI) of nasopharyngitis was similar with different doses (6.05% [2.85%-10.34%] with 5 mg, 6.42% [4.07%-9.27%] with 15 mg, and 5.07% [3.79%-6.53%] with 10 mg) of tirzepatide. Incidence (95% CI) of headache was observed to increase with increasing dose of tirzepatide (4% [1.92%-6.80%], 5.16% [1.10%-11.96%], and 10.71% [4.84%-18.54%]) for the 5-, 10-, and 15-mg dose. Rarely, hypertension, diabetic retinopathy, major adverse cardiovascular events (MACE), and malignant neoplasms were observed in 3 or fewer studies, without any significant association with different doses of tirzepatide (see Table 2).

## Discussion

In our meta-analysis, we have summarized the most precise and up-to-date data on the AEs of tirzepatide using previously published articles. The mean age of the studied population was 59 years with an average duration of diabetes of 10 years and a mean HbA_1c_ of 8.3%. Our systematic review provides details on the incidence rate of each specific AEs at 3 doses of tirzepatide (5, 10, and 15 mg). Besides commonly encountered AEs associated with tirzepatide, our study also evaluated rare AEs associated with tirzepatide including gall bladder and pancreatic AEs. In our study, we found GI AEs to be the most commonly encountered AEs with incidence rates of up to 50% with the 15-mg dose of tirzepatide. For the majority of GI AEs, we found a significant dose-dependent relationship with higher dose being associated with higher incidence rates of AEs. A recent study by Yu et al [27] reported increased total AEs and GI AEs (relative risk [RR] 1.17; *P* = .02) with 10 mg, and increased total AEs (RR 1.10; *P* = .0001) with 15-mg compared to the 5-mg dose of tirzepatide. No statistically significant differences were observed in total AEs and GI AEs for 15-mg compared to 10-mg tirzepatide. In addition, we observed that higher doses of tirzepatide were more likely to be associated with drug discontinuation due to AEs. In particular, the rate of drug discontinuation due to AEs was up to 10% (8%-13%) with the 15-mg dose of tirzepatide. The study by Yu and our colleagues [27] and our study have similar findings for no significant difference in AEs including MACE, hypertension, cancer, and hypoglycemic events with 3 different doses of tirzapatide. In a study by Karagiannis et al [28], the authors found 2-fold higher odds of study drug discontinuation at the highest dose of tirzepatide. Interestingly, our study found that the proportion of serious AEs declined with increasing doses of tirzepatide (7.52%, 7.32%, and 5.37% for the 5-, 10-, and 15-mg doses, respectively) while the incidence rates of cholelithiasis, cholecystitis, and acute pancreatitis were near similar across all 3 doses of tirzepatide (5, 10, and 15 mg). Dutta et al [29], however, reported no significant difference in treatment-emergent AEs and severe AEs in their study comparing tirzepatide with an active control group. Our study also found a higher incidence of hypoglycemia (glucose < 70 mg/dL) with tirzepatide 10 mg, which highlights the need for more careful monitoring during dose titration to avoid hypoglycemia.

Compared to prior meta-analyses assessing the safety and efficacy of tirzepatide [28-31], our study has major differences in the objective and the methodology, making our study unique and noncomparable to previous works. Our meta-regression focused solely on the safety of tirzepatide, with a special focus on establishing the rates of each individual AE at 3 doses of tirzepatide (5, 10, and 15 mg). In the previous meta-analyses, the primary outcome was efficacy (eg, HbA_1c_ reduction, weight reduction), and the safety outcomes were secondary end points of the study. In comparison, our study solely focused on a detailed analysis of safety outcomes. In addition, our study has compiled all the major AEs associated with this agent compared to the previously published meta-analyses. For example, besides commonly encountered AEs associated with tirzepatide, our study also evaluated rare AEs associated with tirzepatide, including gall bladder and pancreatic AEs. The methodology used in our study also differed significantly compared to previously published meta-analyses. For example, compared to the prior work by Karagiannis et al [28], in our study, instead of calculating the odds ratio, we calculated the incidence rates of each individual AE associated with tirzepatide. With the increasing use of tirzepatide beyond the scope of diabetes, knowing the incidence rate of individual AEs would be especially important from a practical standpoint for a broader group of health care providers (such as endocrinologists, primary care physicians, bariatricians, and clinicians treating obesity), who may find this meta-analysis extremely relevant to identify and be vigilant about rare AEs along with commonly reported AEs.

In our meta-analysis, we found the mean age to be 58.9 years, suggesting fewer younger patients in the previous trials. Hence, future studies involving younger patients with diabetes may be necessary to more precisely assess the AEs related to tirzepatide in different age groups. Considering the significant glycemic improvement and weight reduction associated with tirzepatide in previous randomized clinical trials, the use of tirzepatide among patients with or without diabetes is expected to increase in the coming future. Hence, the findings from our study will help clinicians recognize tirzepatide-related AEs at different doses and determine the best treatment strategies for their patients.

Despite its comprehensive methodology, there are several limitations in our study that merit attention. First, in our meta-analysis, individual patient-level variables were unavailable, and analyses were conducted at the study level, which can lead to bias in assessment. Second, the use of meta-analysis to pool published data can be associated with a lack of standardization among the included studies in terms of patient population and outcomes measured, including the severity and definition of each reported AE. Third, most of the included studies reported tirzepatide-related AEs as secondary end points in the original studies. Nonetheless, with careful interpretation of data, the pooled analysis provides valuable information regarding the risk of AEs. Fourth, our meta-analysis included studies published since inception through March 2022 (2018-2022), which is still a short duration to predict long-term AEs, specifically cardiovascular events.

## Conclusion

Our meta-regression provides more precise data on the incidence rate of individual AEs related with tirzepatide use at 3 different doses (5, 10, and 15 mg). We found GI AEs to be the most common AEs associated with tirzepatide use and included nausea, vomiting, dyspepsia, decreased appetite, diarrhea, and constipation. We found tirzepatide to have similar AEs profile as GLP-1 RA. Tirzepatide has been considered a great addition to the arsenal of next-generation medications for T2DM, as evidenced by dose-dependent reduction in HbA_1c_ in prior studies. Our study showed that tirzepatide is safe and has a tolerable AE profile and is expected to play a huge role in the management of T2DM, insulin resistance, and weight loss soon.

## Data Availability

Original data generated and analyzed during this study are included in this published article or in the data repositories listed in “References.”

## Abbreviations

AE: adverse event
ANOVA: analysis of variance
GI: gastrointestinal
GIP: glucose-dependent insulinotropic polypeptide
GLP-1: glucagon-like peptide 1
GLP-1 RA: glucagon-like peptide 1 receptor agonist
HbA_1c_: glycated hemoglobin A_1c_
MACE: major adverse cardiovascular events
T2DM: type 2 diabetes mellitus
